# Addressing housing-related social needs for Medicaid beneficiaries: a qualitative assessment of Maryland’s Medicaid §1115 waiver program

**DOI:** 10.1186/s12913-023-10001-z

**Published:** 2023-09-18

**Authors:** Robert DeGrazia, Abdikarin Abdullahi, MaryAnn Mood, Christin Diehl, Ian Stockwell, Craig Evan Pollack

**Affiliations:** 1 Present Address: Department of Medicine, Virginia Commonwealth University, Richmond, US; 2 Department of Medicine, Johns Hopkins School of Medicine, Baltimore, US; 3 School of Medicine, University of California, San Francisco, US; 4 Johns Hopkins Bloomberg School of Public Health, Baltimore, US; 5 The Hilltop Institute, University of Maryland, Baltimore County, US; 6 Department of Information Systems, University of Maryland Baltimore County, Baltimore, US; 7 Department of Health Policy and Management, Johns Hopkins Bloomberg School of Public Health, Baltimore, US; 8 School of Nursing, Johns Hopkins University, Baltimore, US

**Keywords:** Medicaid, 1115 Waiver, Housing Instability, Homelessness, Housing Programs, Maryland

## Abstract

**Background:**

While health care payers are increasingly considering approaches that help support stable and affordable housing for their beneficiaries, experience with these initiatives is limited. Through its §1115 HealthChoice waiver, Maryland Medicaid has begun experimenting with programs designed to pay for housing and tenancy support/case management services. This study investigates barriers and facilitators to the success of Maryland’s pilot program initiative — Assistance in Community Integration Services (ACIS).

**Methods:**

The study focused on key stakeholders employed by the four Lead Entities that currently participate in the ACIS program. The stakeholders included members of each Lead Entity’s administration, direct service providers, state and local government officials, and case managers from local hospitals. The convenience sample was selected through an initial list of stakeholders and was supplemented using snowball sampling methods. Interviews were audio recorded and turned into transcripts via Otter.ai and then analyzed using NVivo by two independent reviewers.

**Results:**

A total of 23 interviews were conducted between February 2022 and May 2022, representing a broad range of stakeholders across different Maryland geographies. A total of 4 themes were identified through the course of the interviews. Stakeholders identified difficulty finding housing for the target population in a tight housing market, challenges with communication within the program and with its clients, and problems with non-healthcare providers documenting services for reimbursement. At the same time, ACIS was seen as creating opportunities for organizations to work together across siloes in meeting client needs.

**Conclusions:**

The findings of this study helps to highlight Medicaid §1115 waivers as a novel approach to using Medicaid funds to support tenancy-based services, such as ACIS and to improve the lives of individuals while reducing healthcare costs. Implementation of the ACIS program in Maryland has been a resounding success in helping individuals obtain and sustain stable housing. However, continued efforts to align capacity with demand, streamline billing and reimbursement and improve communication with clients and across partners will need to be prioritized. The program also highlights the growing need to address root causes of housing insecurity including the limited supply of affordable housing.

## Background

Although housing stability is increasingly being recognized as an important determinant of health outcomes, an estimated 380,657 individuals in the U.S. experienced unsheltered homelessness according to the 2021 point in time count [1]. Millions more were considered unstably housed, including doubling up on their housing, living in homes with poor physical conditions, or paying more than a third of their income on rent and utilities [2, 3]. The health effects of homelessness and housing instability are wide-ranging, with increased morbidity and mortality, more frequent emergency department visits, longer inpatient hospitalizations, and excess medical expenses compared to the general population [4–15].

Policymakers have increasingly begun implementing initiatives designed to address housing instability as a means towards improving health and decreasing health care costs. In the Medicaid program, Sect. 1115 waivers provide states with an opportunity to introduce programs that are ‘budget neutral’ to the Federal government while better serving Medicaid populations [16]. Six states currently use these waivers to support housing case management and tenancy sustaining services; another 2 states have submitted waiver requests to begin providing housing-related services [17, 18]. Though the states are unable to use Federal funds to pay for rental subsidies, the programs provide a variety of services designed to help individuals who are homeless, at high risk of becoming homeless or institutionalized find housing and remain stably housed.

In their analysis of the first four states to implement housing supports via a Sect. 1115 waiver, including Maryland, Thompson et al. identified several challenges as states were beginning to implement their new initiatives. Their research highlighted concerns about the lack of affordable housing coupled with the limited supply of rental subsidies. Further, they identified a perceived stigma attached to homeless populations based on their social needs and often compounded by structural racism that creates difficulties in finding affordable housing [18]. The stakeholders further identified difficulties working across the housing and healthcare silos, including, in some instances, complex and protracted arrangements to achieve contractual reimbursement mechanisms. The need for a stable workforce to help clients, and concerns around program sustainability were also areas identified as being difficult by stakeholders.

Building on this emerging literature, we sought to use qualitative interviews to identify the facilitators and barriers to program implementation within a single state. Since its start in 2017, Maryland’s Assistance in Community Integration Services (ACIS) program provides housing case management and tenancy support services to individuals who are homeless or at imminent risk of becoming homeless or institutionalized. The focus on the ACIS program provides an opportunity to examine the perspectives from a wide range of stakeholders, identify issues arising further along in the implementation process, and investigate differences in the approach and implementation of the ACIS across jurisdictions within the state.

## Methods

### Setting

The ACIS program, as authorized under the state’s Sect. 1115 waiver, aims to serve “high risk, high utilizing Medicaid enrollees”. The ACIS program provides tenancy-based case management and support services, as well as housing case management [19, 20]. Examples of possible services include, but are not limited to, social services, transportation, landlord communication, and primary care coordination [5–8]. There is also a focus on providing integrated care to the pilot beneficiaries [20]. The waiver did not permit Medicaid funds (either federal dollars or state matching funds) to be used for rental subsidies.

Individuals are eligible if they meet at least one health and one housing criteria. Health criteria are defined as having either repeated incidences of emergency department visits or hospitalizations or two or more chronic conditions. Housing criterion is defined as individuals who are expected to experience homelessness upon release from certain settings or those at imminent risk of institutional placement. Based on the allocated funding, the program has grown to now include a maximum of 900 individuals who can be served by the housing tenancy services of the ACIS program within participating counties across the state.

The program is designed to be overseen by lead local governmental entities (called Lead Entities) that are responsible for the organization of the care delivered in their city or county. Program managers employed by these Lead Entities work with direct service providers employed by community-based organizations (called Participating Entities) that deliver tenancy-based case management and support and receive Medicaid reimbursement. The program managers and direct service providers work in a broader ecosystem that includes health services and specialty mental health agencies, public agencies or departments, or other entities with significant experience serving the target population. The program started in 2017 with three organizations serving as Lead Entities and expanded to four in 2018.

### Stakeholder recruitment and interviews

The study focused on key stakeholders employed by the four Lead Entities that currently participate in the ACIS program, as well as those working at the state level. The stakeholders included members of each Lead Entity’s administration, direct service providers, state and local government officials, and case managers from local hospitals. The convenience sample was selected through an initial list of stakeholders and was supplemented using snowball sampling methods. Potential interviewees were recruited via email and given the option to schedule an in-person, telephone, or video conference visit which was audio recorded.

The interview guide was generated after a review of the literature and was iteratively revised before and during the interview process (see Appendix). The interview guide was designed to identify successes and challenges in the program design and implementation, targeting of beneficiaries, deployment of housing and tenancy services, coordination with healthcare services, and staffing and resources. Interviews lasted approximately one hour, and participants were offered a $40 gift card for their time. Interviews were conducted between February and June 2022.

### Analysis

The interviews were conducted virtually using a video conferencing software and were recorded with the consent of the interviewee. The recorded interviews were transcribed verbatim using Otter.ai software. These transcripts were then reviewed by two members of the research team to correct any errors and then were entered into NVivo to identify major themes. Using a modified grounded theory approach, a codebook was created based on the research questions of interest. Two of the authors (RD and AA) independently coded each transcript and regularly met every two weeks once interviews were completed to compare their coded transcripts and identify emerging themes. After all transcripts were coded, a code book of major themes was created through an iterative process involving the independent coders with feedback from the other authors. These themes were further consolidated at the conclusion of the study to produce four major themes with each major theme having respective subsequent minor themes as well. The Johns Hopkins University and Maryland Department of Health institutional review boards both individually approved the study.

## Results

A total of 23 interviews were conducted between February 2022 and May 2022, representing a broad range of stakeholders across different Maryland geographies (see Table 1). A total of 4 themes were identified through the course of the interviews.

**Table 1:** Stakeholder Characteristics

Characteristics	Number	Percent
Total	23	100.0
Role		
Maryland Department of Health	5	21.7
Lead Entity	6	26.1
Direct Service Provider	12	52.2
Primary Location		
Baltimore City	8	34.8
Cecil/Montgomery County	5	21.7
Prince George County	5	21.7
State Level	5	21.7

### Theme #1: Characteristics of ACIS participants and the tight housing market pose significant barriers; pilot programs implemented novel strategies to try to overcome these barriers

Respondents identified two broad categories of barriers that contextualize the difficulties with finding appropriate housing for clients, and these categories work synergistically to limit access to affordable housing (see Table 2).

**Table 2:** Difficulty finding housing and potential solutions

Theme	Subtheme	Representative quotation
Difficulty finding housing	Related to client circumstances	“Even if the housing is available, these clients come in with a lot of evictions in their past. Maybe they had…bad credit…some of them just apply and apply and apply. One time it took us a year to find a unit for one of our participants…”
	Housing-market conditions	“I would argue across the [jurisdiction] [and] the state… there are times where even if a provider can get section eight housing vouchers [now called Housing Choice Vouchers] doesn't mean you can find an apartment owner or a housing provider who's willing to accept the section eight housing voucher. It’s a fight for limited resources…”
Novel solutions for housing	Investment in Affordable and Accessible Housing	“So, both [Direct Service Provider] and [jurisdiction] recently hired an expert in housing development because they have a strategic plan to develop affordable housing themselves.”
	Landlord Risk Mitigation	“…if a client, because some of them have mental challenges, breaks something, we will fix it. So, the cost is not on the landlord.”

The first category of barriers involves characteristics related to the participant’s background. One respondent stated that clients sometimes are perceived to be “undesirable” by landlords due to a range of issues related to being homeless or at risk of homelessness, including those due to prior rental histories, credit scores, and comorbid conditions. Interviewees also noted sigma against individuals without housing as a related barrier for finding housing.

The second category of barriers refers to issues with local housing affordability and supply. Universally, program leaders identified enormous increases in rental prices as an obstacle. Because ACIS does not offer direct rental assistance, high rents remain a significant concern. Even when individuals were able to receive housing vouchers, they were, at times, unable to find affordable housingThis was further compounded by the fact that some landlords were hesitant to accept housing vouchers, despite the fact they are legally required to do so. One interviewee described the broader context of finding housing as, “…obviously rental prices are going up especially, you know, in [county name]. That seems to be a difficulty for most of the agencies… is finding reasonably priced rental-housing for clients [and] with inflation, the way it's going, that's going to be a continuous obstacle to try to tackle.”

Respondents reported that local jurisdictions have adopted independentstrategies to address concerns regarding participant rental histories and backgrounds and worked to form relationships with landlords to navigate existing biases against the target population.

This has been augmented by measures to decrease the degree of risk for landlords, such as through the creation of risk mitigation funds and substantial security deposits. These measures provide landlords with a financial commitment from the pilot programs that will cover property damage, which has successfully increased the willingness of these property managers to engage with the ACIS program.

### Theme #2: ACIS leveraged existing resources to work across siloes

The ACIS program was able to meet its intended target of bringing together stakeholders at the local and state levels to reduce the siloing of services for this vulnerable population and optimize care coordination. This required leveraging existing resources and then working to coordinate across those resources (see Table 3). Communities had different levels of resources to draw upon—including personnel within the county program manager’s office, community organizations with expertise working with the target population, and services already tailored towards the needs of the target population. Outside of the county program managers and direct service providers, interviewees discussed other key stakeholders such as housing authorities, hospitals, businesses, departments of social services, behavioral health authorities, and police departments. The presence or absence of these resources led to some heterogeneity in the stakeholders involved in the ACIS program within local jurisdictions, but there was a universal emphasis on identifying and partnering with stakeholders to better coordinate care.

**Table 3:** Leveraging existing resources and working across siloes

Theme	Subtheme	Representative Quotation
Existing infrastructure	Leveraging existing infrastructure	“I think, the way we have it implemented within our continuum, it partners and bodes well with our current existing permanent supportive housing program. So, it just provides an extra layer of supportive services to that already supported housing program and makes it much easier to implement. Additionally, we have some of the [infra]structure within the county to be able to pull from so that we can support the program, I think at a higher level which has made it successful.”
Stakeholders and collaboration	Regular meetings	“In our [jurisdiction], we have a quarterly meeting called the intra-agency meeting on homelessness, and that involves all the stakeholders in the [jurisdiction] who work with the homeless, it deals with the jails, schools, housing programs, etc.”
	Improved care coordination	“The stakeholders are very talented and [make up an] instrumentally competent community of committed individuals and it takes an immense amount of cross-disciplinary expertise, an immense amount of prior personal experience as well as professional experience to commit, the energy [and] the focus, that it takes to effectively provide integrated health and housing services for the most vulnerable.”

Interviewees noted several strategies designed to coordinate care for their clients and work towards a more integrated system. Stakeholders would often meet to discuss how to best serve participants, including the barriers they faced towards housing. For systems change, stakeholders noted the need for broad engagement to promote cross-communication, share best practices, and improve client outcomes.

Outside of engagement strategies tailored towards coordinating across silos, stakeholders commented on the importance of expertise that spans the housing and health sectors. This was facilitated by the presence of stakeholders and community-based service providers with expertise in housing and health services. Interviewees expressed a desire for stakeholders to ideally have expertise in both domains. The ACIS program, therefore, directly addressed the link between housing and health by promoting interdisciplinary expertise to improve outcomes for the target population.

### Theme #3: Improving communication was viewed as critical

Challenges with communication both within the ACIS program and with its clients were often raised as a barrier to effective program implementation and sustainability (see Table 4).

**Table 4:** Communication challenges within the ACIS program and with its clients

Theme	Subtheme	Representative Quotation
Communication with ACIS Program	Challenges with follow-up after referrals were made	“…my staff will refer patients to the program, but then we don't necessarily get any feedback, regarding what the outcomes have been. So, Mr. Jones, for example, if we identified Mr. Jones in the emergency department and refer him to the program, we have no idea if Mr. Jones has housing [or] if he's more stable.”
Communication with ACIS Participants	Challenges in maintaining contact with clients	“The usual [challenges] that go along with serving the homeless population, which is maintaining contact, especially when there's significant substance use present.”
	Challenges with communication-related to COVID-19	“It could be that they're symptomatic and they're paranoid and they won't answer the door. [They] could have had different service coordinators, somebody could have left, they talk about retention. So, if you have a relationship with a service coordinator, a new one comes, they may be resistant to meeting with folks [and] the face-to-face clearly went down during COVID.”

Direct service providers and community stakeholders that typically would refer clients to the ACIS program noted in interviews that their communication with county program managers was suboptimal and they frequently acknowledged a lack of follow-up after referring a client. As one referring provider said, “Once we send them, it's very rare that we get specific follow-up regarding that [referral], unless, anecdotally, a patient might come back to the hospital who may say that [the referral] didn't pan out or I'm still homeless, but there hasn't been or there isn't any mechanism in place for us to appropriately track that.”

A recurring sub-theme that also emerged is difficulty with participant retention, primarily involving the loss of communication with clients. This is likely, at least in part, due to the population they are attempting to serve as many homeless or housing insecure individuals have inconsistent access to phones and/or permanent addresses. Further, direct service providers explained that communication challenges also arise due to the higher rates of untreated mental health conditions and/or substance use within the target population.

The communication issue was further exacerbated by the ongoing COVID-19 pandemic. Many service providers noted difficulties establishing meaningful connections with their clients without being able to meet face-to-face. One described, “I think one challenge was…building relationships with clients that were new to us, over the phone, and all the difficulties that come along with that. There's a lot to be said, I think, for nonverbal communication, and just meeting face to face with folks…”.

### Theme #4: Collecting data was seen as a key challenge in program implementation

A fourth theme was around challenges in documenting services delivered to receive reimbursement and to demonstrate programs effectiveness. In general, the four counties that operate within the ACIS program are required to pay half of the program costs through local matching funds (which count towards the state-required funding) while the other half come from the federal government via Medicaid. In one of Maryland’s jurisdictions, local hospital systems contribute money for the local matching funds. To receive reimbursement from both the local and federal funds, direct service providers are required to provide and document a minimum number of services that were delivered to a client each month.

A considerable proportion of interviewees worried that direct service providers—who often worked in community-based organizations and were not healthcare-based—were not as familiar with the documentation requirements (Table 5). Compounding concerns, many noted that the data was collected using spreadsheets in ways that were inefficient and time consuming. Interviewees stated that some direct service providers were unable to keep up with these requirements and subsequently had to cease their participation in ACIS. As one interviewee said, “One of the challenges that our [direct service providers] complain about is the data and how much we must collect and how it is collected. If there was a way that all jurisdictions collected data the same way, in the same system or platform, it would be a lot easier.”

**Table 5:** Stakeholders expressed concern over data collection for billing

Theme	Subtheme	Representative Quotation
Data collection challenges	Difficulties with data collection affecting direct service providers	“I think for, some [service providers] that are not as familiar with…the concepts of billing Medicaid. I mean it is not that different from any other insurance company [where] you must submit the claim. You must get reimbursement and you must justify you know, the reimbursement so that is where the data comes in.”
	Difficulties with data collection affecting reimbursement	“The challenging part was when providers did not meet the three services [required for reimbursement],,,some of the providers did not document it right.”
Hospital involvement	Hospital concerns over the need for data	“….as stakeholders who are paying into this, we want to know that the community members are being supported and what’s happening with the dollars.”
	Hospital concerns over long-term funding	“If you’re just replacing a government dollar with a hospital dollar, we’re not making a difference. I’m not saying hospitals don’t have a role supporting these kinds of programs but there needs to be some sort of maintenance of effort on the government side or some sort of corollary investment.”

Despite continued efforts to train direct service providers on appropriate documentation, this problem is further exacerbated by the continued staffing turnover. Discussions also focused on the difficulty using Excel as a manual data collection tool and cited differences in workflow between healthcare-based (i.e., hospitals) and community-based organizations as potential contributing factors.

Related to the need for data collection for billing, respondents also noted the challenges of data collection to demonstrate improved outcomes. Demonstrated improved outcomes was not a requirement of their receiving funding for the services they delivered but was seen as important for continued investment in the program. Conversations with hospital administrators acknowledged the desire to understand how services were being delivered and whether there was a return on their investment, given that they were contributing to the local matching funds in one Maryland jurisdiction. As one indicated, “….as stakeholders who are paying into this, we want to know that the community members are being supported and what’s happening with the dollars.”

Further, the hospital administrator’s comments noted concern in the current funding structure in which local governments may be relying too heavily on them as a permanent funding mechanism and the implications this may have for healthcare costs in general. As one hospital executive said, “Even if hospitals are investing in housing, but the government is not making an equal increase in housing, then we’re never going to move the needle on these big social determinants of health.”

## Discussion

With a growing understanding of the ways that housing insecurity contributes to high healthcare costs, identifying payment mechanisms and developing best practices designed to address housing-related social needs has received increasing attention. Maryland’s ACIS program provides an important example of a Medicaid §1115 waiver focused on reducing the cost and burden of high healthcare spending among individuals who are insecurely housed. The results of the interviews with key informants underscore strategies that are contributing to the ongoing success of the program—including ways in which the program has leveraged existing community resources and improved coordination across these resources—as well as barriers to its implementation—including the tight housing market and difficulties with communication and data collection. These findings provide an important template as other states, payers, and health care systems move to meet patients’ social needs.

This study reinforces the work by Thompson et al., showing that many of the challenges that existed during the early implementation of housing-focused programs persist in longer-term follow-up, hampering program efficiency and sustainability. While it is not surprising that issues related to the tight housing market continue, the difficulties with reimbursement and program sustainability remained salient for key informants. These findings highlight areas that would benefit from planning as states consider programs designed to utilize Medicaid funds to support housing and tenancy-based services.

Program success stemmed from flexibility in leveraging existing resources across jurisdictions within the state. Stakeholders recognized that efficiently using the resources provided by the §1115 waiver—which required matching funds—necessitated coordination amongst stakeholders already working with the target population. Though not specifically discussed by stakeholders, this alignment of resources and matching of funds likely benefited from the state’s unique healthcare financing. Under Maryland’s previous model, the All-Payer Medicare Model Contract, hospitals received global budgets, which are largely based on historical hospital spending. These budgets, as opposed to more traditional fee-for-service models are designed to align hospital incentives to reduce expenditures and increase quality for the population it serves. Its successor program, the Total Cost of Care Model, came into effect in 2019 and seeks to limit per capita expenditures through population-based hospital payments, advances in primary care, and other avenues. The model established the Statewide Integrated Health Improvement Strategy through which state and industry stakeholders identified housing as the key social determinant of health that would drive success toward the population health goals [21]. Also under the Total Cost of Care Model, Maryland is working with hospitals to determine what hospitals should do with retained revenue under their global budgets. Supporting housing-related interventions has arisen as an idea of where to channel those retained revenues with discussions ongoing, potentially helping sustain §1115 waiver matching funds [21]. Existing healthcare infrastructure and policy priorities in other states may similarly influence the success of Medicaid funded housing supports.

Many interviewees described the broader difficulty in identifying safe and affordable housing generally, a problem made more challenging for individuals whom landlords may deem ‘risky’, including those with substance use or mental health disorders and prior evictions. The findings here suggest that some tenancy support services were useful in helping overcome barriers, for example, the development of a landlord risk mitigation fund to help address potential damage done to housing units. The size and funding model of risk mitigation funds varied across lead entities and was not discussed in detail by respondents.

Other problems, however, such as the limited supply of affordable housing and limited rental assistance require more structural changes. Many of these issues are largely outside the traditional purview of healthcare (Medicaid funds are not permitted for the construction of housing units) and instead necessitate broader investments at the federal-, state-, and local-levels. For example, at the federal level, there has been policy attention towards increasing the supply of federal rental assistance which currently serves approximately a quarter of eligible households [22]. States are currently experimenting with ways to accomplish the goal of increasing the supply of affordable housing, which is a key driver of housing instability and homelessness. These initiatives range from programs that convert hotels and motels into permanent supportive housing to initiatives that seek to change zoning requirements to allow more housing to be built in high demand areas [23–26].

Maryland’s §1115 waiver did not permit Medicaid funds to be used to pay rent. The Centers for Medicare and Medicaid Services recently released guidance indicating that rent or temporary housing may be considered under §1115 waiver applications for individuals who meet certain criteria (i.e., transitioning out of institutional care or congregate settings, individuals who are homeless, youth transitioning out of the child welfare system) [27]. Given the difficulties stakeholders observed in securing and funding housing, this flexibility may offer states who choose to apply promising opportunities.

Data collection to support reimbursement and to determine program effectiveness was identified as a critical challenge to the continued funding and success of the ACIS program. Medicaid billing requires providers to document the delivered services in a manner fundamentally different from their routines. This challenge was compounded by using spreadsheets, which many direct service providers had limited experience with, and by ongoing staff turnover. The findings extend the work by Thompson and colleagues by suggesting that problems stem from not only setting up agreements and reimbursement mechanisms but also by how they play out in practice. Recognizing these challenges and working to design policies that align with community-based provider workflow is critical as these programs expand.

Finally, communication was also noted to be a recurring theme. This aligns with prior work demonstrating the difficulties of working with a hard-to-reach population by virtue of their housing insecurity and limited financial resources. These challenges were exacerbated by the move to virtual communication strategies necessitated by the COVID-19 pandemic. Concerns over communication were not only limited to interactions with clients and stakeholders but also between health care organizations making referrals and service providers. Working to improve communication is important in continuing to generate referrals and developing on-going institutional support.

This study had several limitations. First, while we sought to include a diverse range of perspectives to generate broad themes and achieved thematic saturation, the themes identified in this study might not capture the full range of perspectives across the state. Second, though individuals were speaking from their professional capacity, interviews may be subject to recall and social desirability bias. Third, the project does not capture the perspectives of individuals who were homeless or unstably housed. Their perspective on whether and to what extent the program addressed their needs and how it could be improved is a crucial next step. Further, the study was not able to recruit landlords, who may also have a unique and different perspective of the ACIS program. Understanding the perspectives of landlords in the low-income rental market is a growing area of inquiry and is necessary to address barriers. Finally, the study focused on a single state which expanded its Medicaid under the Affordable Care Act and has unique healthcare financing under the Total Cost of Care Model. In depth case studies of a single area are important in generating new knowledge which can be tested in other jurisdictions and settings.

## Conclusion

Medicaid §1115 waivers allow novel approaches to use Medicaid funds to support tenancy-based services, such as ACIS, to improve the lives of individuals while reducing healthcare costs. Implementation of the ACIS program in Maryland has been a resounding success in helping individuals obtain and sustain stable housing. However, continued efforts to align capacity with demand, streamline billing and reimbursement and improve communication with clients and across partners will need to be prioritized. The program also highlights the growing need to address root causes of housing insecurity including the limited supply of affordable housing.

## Data Availability

The datasets used and/or analyzed during the current study are available from the corresponding author on reasonable request.

## Appendix 1: Interview question guide

### Role

1. What has been your role in the development and/or implementation of the ACIS program?

### Program design and implementation

2. In your view, what are the key factors in the success of getting the ACIS program to this point? In what ways has ACIS been successful?
3. [Administrative Question] In your view, what have been the most difficult challenges in getting the ACIS pilot program off the ground and operating? What challenges were more easily met? What challenges have been the hardest to overcome?

### Services and collaboration

4. What group(s) of Medicaid enrollees have you primarily targeted for housing assistance? Where does your effort to identify and serve these beneficiaries currently stand? What challenges have you encountered in targeting and serving these enrollees? Have you changed the focus on your target population during the implementation process?
5. Housing support waivers to aid those at risk of homelessness or avoidable institutionalization require cooperation between stakeholders in the health care, housing, and other sectors. What strategies have been pursued to foster such collaboration?
  - Who are the key stakeholders involved?
  - What strategies do you think are most important to continue moving forward?
  - Are there strategies that have not been attempted yet and that might be useful?

### Housing tenancy services

6. What particular tenancy sustaining services have you offered?
  - What implementation challenges have you faced so far?
  - How successful have clients been in engaging with these tenancy sustaining services?
  - What services have been particularly helpful in helping families with finding housing? What services have been less useful?
  - What challenges, if any, have you encountered with these services?
  - What advice would you have to make these more successful?

### Healthcare

7. In your opinion, what has been the most important factor(s) to consider when supporting a client’s health and need for healthcare?
8. What are the most common barriers clients face when addressing their health care needs?
9. On average, how long do you think participants have stayed in the ACIS program, and what are the reasons people have left the program so far? What efforts, if any, will be made to help beneficiaries build independence and exit the housing support services when, and if, they no longer need the benefit?

### Staffing and resources

10. What additional staffing resources are needed to support the running of the program?
11. What additional financial resources, if any, are needed to support the running of the program?
12. Are there local issues with the cost, supply, or other characteristics of housing that affect the program?
13. Other states may decide to pursue Medicaid waivers with housing support. Based on your experience in developing and getting these initiatives up and running, what advice would you offer?
14. Are there issues we didn’t discuss that you think are important for understanding the experience with the Medicaid housing-support waivers?

### Closing

15. As we go forward with the project, is there anyone whom you would suggest we contact who could further inform us of the ACIS program’s development and early implementation?

## Abbreviations

ACIS: Assistance in Community Integration Services
RD: Author initials
AA: Author initials
